# Perioperative management of diabetic foot

**DOI:** 10.3389/fphar.2014.00091

**Published:** 2014-05-01

**Authors:** Nune Soghomonyan

**Affiliations:** Department of General Surgery, Kanaker-Zeytun Medical Center, Armenian Association of Diabetic FootYerevan, Armenia

**Keywords:** diabetic foot, diabetic ulcers, diabetes mellitus, wound care

Currently, 382 million people, or up to 8.8% of total world population, live with diabetes mellitus (International Diabetes Federation, 2013). By 2035, these numbers will reach 592 million acquiring the characteristics of an epidemic, similar to other lifestyle-related epidemics including obesity, cardiovascular diseases and others. Approximately 80% people with diabetes live in low income countries. Specially alarming is the spread of type 2 diabetes (T2D) among children including the younger age group.

Annually, nearly 1 million people undergo high lower extremity amputations because of complications of diabetes. In other words, 1 amputation is being performed worldwide every 20–30 s, and the vast majority of these amputations are in patients suffering from diabetic foot ulcers (International Diabetes Federation, 2013).

Classically, the development of diabetic foot ulcer is promoted by the diabetic polyneuropathy and frequent traumatization of the insensitive foot with further bacterial or fungal contamination of the wound, peripheral arterial occlusive disease, and aggressive artheriosclerosis observed commonly during persistent and poorly controlled hyperglycemia (Vlassara and Striker, 2013). The diabetic foot ulcers themselves are regarded as precursors of typically painless cardiovascular or cerebrovascular major accidents, thus their effective therapy mandates a multidisciplinary team approach.

Another major threat of lower limb loss is the complexity of foot deformities in the already autosympathectomized osteoarthropatic diabetic foot with habitually intact arterial inflow. Subluxations, fractures, swelling, and inflammation of soft tissues in combination with reduced sensitivity and loss of pain perception may lead to disruption of skin integrity and development of local infectious and generalized septic complications. The degree of damage in diabetic foot is directly related to the socio-economic status and quality of diabetic foot care. A quarter of diabetic foot ulcers eventually result in minor or major amputations of the affected limb. Cases with lack of multifaceted treatment and resultant higher level amputations compose the high risk group for perioperative mortality and contralateral lower limb loss within 3 years. The 5-year risk of death in this group of patients surpasses cancer mortality (International Diabetes Federation, 2013).

The hyperglycemia is considered a clue factor for tissue damage in diabetes (Nandi and Poretsky, 2013). Multiple mechanisms for diabetes development have been proposed. However, the principal factor determining the disease progression is severity and duration of hyperglycemia which, if sustained, causes retinopathy, neuropathy, ischemia, supports local infections, and other complications of advanced diabetes.

Pathophysiological mechanisms predisposing to formation of tissue alteration include generation of advanced glycation end-products (AGEs) or glycotoxins and action of advanced lipoxidation products (ALEs) either of dietary origin or from intracellular sources (Gabbay et al., 2014). As potent prooxidants, they increase the intracellular oxidative stress, deplete the anti-oxidant reserve, and induce an inflammatory reaction. In addition, AGEs appear to be directly cytotoxic to the pancreatic beta-cells. Besides undergoing direct reduction in lysosomes, AGEs undergo filtration in kidneys, which makes these patients extremely vulnerable to kidney dysfunction. Impaired renal function markedly increases the overall oxidative stress in the whole organism including the vital organs. All these detrimental effects significantly impact the course of diabetic foot.

The perioperative period in these patients is characterized by superimposition of procedure-related additional risks and adverse factors on the already existing hyperglycemia, infection and tissue alteration. Perioperative management of patients undergoing procedures to treat the diabetic foot requires preoperative stabilization of the vital organs, infection control, restoring of the acid-base balance, correction of electrolytes, colloid-osmotic pressure, and blood volume. It is crucial to tightly control the blood glucose level. Many times, intraoperative insulin infusion under control of blood glucose level is justified to prevent hyperglycemia and improve the outcome.

Reduced antioxidant defenses can be restored by restriction of dietary AGEs and ALEs (e.g. red meat) and prescription of drugs with antioxidant properties (Kang et al., 1998; Corathers et al., 2013; Kim and Steinberg, 2013). Gabbay et al. (2014) hypothesize that the tissue blood flow in diabetic foot is rerouted through the metarteriole thoroughfare channel, bypassing the exchange capillaries (Gabbay et al., 2014). This disables the nutrient and oxygen exchange in tissues and results in local hypoxia.

Diabetic patients typically suffer from symmetric bilateral tibial stenoses with relatively spared femoral and pedal arteries. The development of macrovascular disorders suggests symptomatic peripheral arterial disease and possibility of effective restoration of the arterial inflow in the large vessels (Jindal et al., 2013). However, successful resumption of blood flow in major vessels does not necessarily correlate with adequate perfusion of the tissues because of altered capillary circulation and neuropathic vascular tone dysregulation (Zambouri, 2007; Šponer et al., 2013). In these cases the blood rheology improvement turns especially important. Controlled anticoagulation is highly recommended with perioperative INR 1,5 achievement, which is considered to be generally safe for intra-postoperative invasive surgical major hemorrhagic complications, meantime significantly improves the perfusion of myocardium, brain, kidneys, extremities (Zambouri, 2007; Jindal et al., 2013).

The administration of intravenous (IV) heparin or subcutaneous (SC) low-molecular-weight heparin (LMWH) should be individualized. It takes approximately 3 days for the INR to reach 2.0 once oral anticoagulant is restarted postoperatively (Frykberg et al., 2006; Zambouri, 2007).

Frequently, therapy of the related comorbidities including the underlying chronic or acute disseminated intravascular coagulation allows for more significant improvement of microcirculation than poorly justified aggressive invasive vascular interventions (O'Reilly et al., 2011).

Further assessment and treatment of concomitant acute regional septic arteriitis and phlebitis is paramount, as infection control will help to reverse the microcirculatory impairment (Corathers et al., 2013).

Severe diabetic neuropathy is the principal contributor to development of neuropathic osteoarthropathy of Charcot—a rare limb-threatening foot deformity, characterized by presence of pulsatile pedal arteries, development of complex and diverse fractures, subluxations, and reactive soft tissue swelling after a minor and usually underestimated trauma. The disease is triggered in susceptible individuals through a process of uncontrolled inflammation leading to osteolysis, progressive fractures and articular malpositioning due to joint subluxations and dislocations (Šponer et al., 2013). Neuropathy and related local anesthesia predispose to chronic trauma of the foot, while the autonomic neuropathy results in increased regional blood flow with resultant soft tissue edema and local osteoporosis (Lechleitner et al., 2012; Levitt et al., 2013).

In the acute phase, avoidance of weight bearing is a prerequisite for prevention of mid-foot collapse. Even after consolidation, abnormal bony prominences may cause ulcerations and foot infections. Surgical reconstruction is indicated if the deformity can't be adequately managed by shoe modifications and bracing.

Generalized and local infections are risk factors which may prevent recovery, exclude favorable outcome, and increase the rate of limb amputations in diabetic foot patients (Wu et al., 2007; Levitt et al., 2013; Šponer et al., 2013). Treatment is started by the administration of wide spectrum antibiotics covering both aerobic/anaerobic spectrum and continued by specimen sensitive spectrum. Diabetic ulcers are usually colonized by mixed bacterial associations. Ischemic ulcers frequently are colonized by anaerobes. Preferred are IV Moxifloxacin or third generation cephalosporine/metronidazole combination. Antifungal treatment is initiated immediately orally or IV. Perioperative wound regeneration, graft patency, and particularly affected limb predetermination as well as postoperative morbidity and mortality largely is depending on effectiveness of antibacterial prevention/treatment. A compromised local and general immune response predisposes to development of infections *per se*, however, the risk increases significantly in patients with underlying diabetic polyneuropathy and chronic tissue hypoxia/ischemia (Lipsky et al., 2012).

Approaches to surgical and pharmacological management of septic wounds are well established. The therapy of infected diabetic foot requires meticulous surgical debridement and decompression of viable tissues, mechanical, and pharmacological destruction of the microbial biofilms in combination with targeted systemic and local antibacterial therapy. In addition to the local therapeutic measures, systemic correction of cardiovascular, respiratory, renal, and blood coagulation functions is mandatory (Frykberg et al., 2006; Zayed et al., 2009; O'Reilly et al., 2011).

In conclusion, patients undergoing surgery for diabetic foot are characterized with disorders of local and systemic functions. The final surgical outcome will depend on perioperative correction and stabilization of organ-systems, tight control of blood glucose levels and aggressive infection control.

## Conflict of interest statement

The author declares that the research was conducted in the absence of any commercial or financial relationships that could be construed as a potential conflict of interest.

## References

[B1] Corathers S. D. Peavie S. Salehi M. (2013). Complications of diabetes therapy. Endocrinol. Metab. Clin. North Am. 42, 947–970 10.1016/j.ecl.2013.06.005 24286957 PMC6533625

[B2] Frykberg R. G. Zgonis T. Armstrong D. G. Driver V. R. Giurini J. M. Kravitz S. R. (2006). Diabetic foot disorders: a clinical practice guideline (2006 Revision). J. Foot Ankle Surg. S-2–S-66 10.1016/S1067-2516(07)60001-5 17280936

[B3] Gabbay I. E. Gabbay M. Gabbay U. (2014). Diabetic foot cellular hypoxia may be due to capillary shunting—A novel.hypothesis. Med. Hypotheses. 82, 57–59 10.1016/j.mehy.2013.11.005 24280559

[B4] International Diabetes Federation. (2013). Available online at: http://www.idf.org/diabetesatlas (Accessed February 25, 2014).

[B5] Jindal A. Garcia-Touza M. Jindal N. Whaley-Connell A. Sowers J. R. (2013). Diabetic kidney disease and the cardiorenal syndrome: old disease, new perspectives. Endocrinol. Metab. Clin. North Am. 42, 789–808 10.1016/j.ecl.2013.06.002 24286950 PMC4251585

[B6] Kang M. Y. Tsuchiya M. Packer L. Manabe M. (1998). *In vitro* study on antioxidant potential of various drugs used in the perioperative period. Acta Anaesthesiol. Scand. 42, 4–12 10.1111/j.1399-6576.1998.tb05073.x 9527743

[B7] Kim P. J. Steinberg J. S. (2013). Complications of the diabetic foot. Endocrinol. Metab. Clin. North Am. 42, 833–847 10.1016/j.ecl.2013.08.002 24286952

[B8] Lechleitner M. Abrahamian H. Francesconi M. (2012). Diabetischer Fuß. Wien Klin Wochenschr. 124(Suppl. 2), 39–41 10.1007/s00508-012-0264-4 23250452

[B9] Levitt B. A. Stapleton J. J. Zgonis T. (2013). Diabetic Lisfranc fracture-dislocations and charcot neuroarthropathy. Clin. Podiatr. Med. Surg. 30, 257–263 10.1016/j.cpm.2013.01.002 23465814

[B10] Lipsky B. A. Berendt A. R. Cornia P. B. Pile J. C. Peters E. J. Armstrong D. G. (2012). 2012 Infectious diseases society of america clinical practice guideline for the diagnosis and treatment of diabetic foot infections. Clin. Infect. Dis. 54, 132–173 10.1093/cid/cis346 22619242

[B11] Nandi A. Poretsky L. (2013). Diabetes and the female reproductive system. Endocrinol. Metab. Clin. North Am. 42, 915–946 10.1016/j.ecl.2013.07.007 24286956

[B12] O'Reilly D. Linden R. Fedorko L. Tarride J. E. Jones W. G. Bowen J. M. (2011). A prospective, double-blind, randomized, controlled clinical trial comparing standard wound care with adjunctive hyperbaric oxygen therapy (HBOT) to standard wound care only for the treatment of chronic, non-healing ulcers of the lower limb in patients with diabetes mellitus: a study protocol. Trials 12:69 10.1186/1745-6215-12-69 21385365 PMC3061927

[B13] Šponer P. Kucera T. Brtková J. Srot J. (2013). The management of Charcot midfoot deformities in diabetic patients. Acta Medica (Hradec Kralove). 56, 3–8 23909047 10.14712/18059694.2014.30

[B14] Vlassara H. Striker G. E. (2013). Advanced Glycation Endproducts in diabetes and diabetic complications. Endocrinol. Metab. Clin. N. Am. 42, 697–719 10.1016/j.ecl.2013.07.005 24286947

[B15] Wu S. C. Driver V. R. Wrobel J. S. Armstrong D. G. (2007). Foot ulcers in the diabetic patient, Prevention and Treatment. Vasc. Health risk manag. 3, 65–76 17583176 PMC1994045

[B16] Zambouri A. (2007). Preoperative evaluation and preparation for anesthesia and surgery. Hippokratia 11, 13–21 19582171 PMC2464262

[B17] Zayed H. Halawa M. Maillardet L. Sidhu P. S. Edmonds M. Rashid H. (2009). Improving limb salvage rate in diabetic patients with critical leg ischaemia using a multidisciplinary approach. Int. J. Clin. Pract. 63, 855–858 10.1111/j.1742-1241.2007.01608.x 18248395

